# Effect of present versus previous smoking on non-invasive haemodynamics

**DOI:** 10.1038/s41598-018-31904-6

**Published:** 2018-09-11

**Authors:** Manoj Kumar Choudhary, Arttu Eräranta, Antti J. Tikkakoski, Heidi Bouquin, Elina J. Hautaniemi, Mika Kähönen, Kalle Sipilä, Jukka Mustonen, Ilkka Pörsti

**Affiliations:** 1 Faculty of Medicine and Life Sciences, University of Tampere, Tampere, Finland; 2 Department of Clinical Physiology, Tampere University Hospital, Tampere, Finland; 3 Department of Internal Medicine, Tampere University Hospital, Tampere, Finland

## Abstract

We examined cardiovascular function in 637 volunteers (19–72 years) without antihypertensive medication in never smokers (n = 365), present smokers (n = 81) and previous smokers (n = 191, median abstinence 10 years). Haemodynamics during passive head-up tilt were recorded using whole-body impedance cardiography and radial pulse wave analysis. Results were adjusted for age, sex, body mass index, LDL cholesterol and alcohol use. Systolic and diastolic blood pressure, heart rate, and pulse wave velocity were not different between the groups. Supine aortic reflection times did not differ, while upright values were shorter in present versus previous smokers (p = 0.04). Heart rate adjusted augmentation index was increased in the supine position in present smokers versus controls (p = 0.045), and in present (p < 0.001) and previous (p = 0.031) smokers versus controls in the upright position. Supine and upright cardiac output was higher (p ≤ 0.016) and systemic vascular resistance lower (p ≤ 0.001) in present versus previous smokers. In spite of the long abstinence, in the upright position previous smokers had lower cardiac output (p = 0.032) and higher systemic vascular resistance (p = 0.014) than never smokers. In the absence of differences in blood pressure and arterial stiffness, present smokers presented with hyperdynamic circulation and enhanced wave reflection compared with previous smokers.

## Introduction

Cigarette smoking is one of the most important preventable risk factors for mortality in the Western world^1,2^, accounting for more than 5 million premature deaths globally per year^3^. Smoking is also the second most common cause for cardiovascular disease (CVD) after elevated blood pressure (BP)^4^. According to World Health Organisation more than one billion people smoke and the prevalence is continuously rising^5^. Cardiovascular deaths account for >54% of all deaths worldwide, and more than 10% of these deaths are attributed to smoking^6^.

Smoking predisposes to the progression of atherosclerosis, shown as increased arterial intima-media thickness (IMT)^7^, and higher prevalence of atherosclerotic plaques in autopsy studies^8^. In a study with 10,914 patients the progression of atherosclerosis in current smokers was increased by 50% versus non-smokers, documented using measurements of IMT in the carotid artery^7^. Smoking is also associated with adverse effects on serum lipids^9,10^, insulin resistance^11^, and activation of the sympathetic nervous system^12^. Carbon monoxide in the inhaled cigarette smoke increases the levels of carboxyhemoglobin, the proportion of which can exceed 7.5% in smokers, while the average level in non-smokers is 0.32%^13^. Although very high levels are uncommon, symptomatic effects may occur at carboxyhemoglobin levels of 2.5% or more^13^.

Controversial reports have been published about the effect of smoking on BP^14–17^. Gropelli *et al*. reported that smoking causes an acute 15–20 mmHg rise in systolic BP, but the effect starts declining after 10 minutes and can be missed if BP is measured more than 30 minutes after smoking^15^. Some previous reports found that male smokers have increased BP^16^. In contrast, some studies reported that smokers have lower BP than non-smokers^17,18^. The putative reduction of BP in smokers may be related to lower body weight, while previous smokers often have higher body weight and increased BP versus never smokers^19^. The vasodilator effect of the nicotine metabolite cotinine may contribute to the reduction of BP in current smokers^20^.

Increased arterial stiffness is an independent predictor of CVD^21^. Many studies have reported that chronic smoking is a risk factor for increased arterial stiffness, however, a number of investigations have not found differences in arterial stiffness between smokers and never smokers^22,23^. Higher augmentation index (AIx), a marker of wave reflections, has been found to be associated with smoking in several studies^14,24–27^. Argacha *et al*. reported that acute smoking increases AIx^14^, while 1-hour exposure to passive smoking was found to increase AIx by 15.7 percentage points^25^. Polonia *et al*. found that AIx was reduced by about 9 percentage points in subjects who stopped smoking for 6 months, whereas there was an increase of 1.7 percentage points in those who continued smoking^28^.

Altogether, the effects of smoking on BP and arterial stiffness remain controversial, while increased AIx has been documented in many studies but the underlying mechanisms are not well understood. Here we examined putative differences in haemodynamics between present, previous, and never smokers. To gain insight about the function of the cardiovascular system in the study groups a passive head-up tilt was included in the study protocol.

## Methods

### Participants

All participants were from an ongoing study, the primary aim of which is to examine haemodynamics in subjects with primary and secondary hypertension versus normotensive controls, in both supine and upright positions (DYNAMIC study; ClinicalTrails.gov identifier NCT01742702). The total number of all enrolled subjects is 1349. The exclusion criteria for the present study were: use of BP-lowering or other medication with direct cardiovascular influences, secondary hypertension, and history of coronary artery disease, stroke, heart failure, valvular heart disease, diabetes, chronic kidney disease, alcohol or substance abuse, psychiatric illnesses, or other heart rhythm than sinus.

The participants were enrolled by announcements from the personnel and patients treated at Tampere University Hospital, personnel of the University of Tampere, and clients of the Varala Sports Institute and local occupational health care providers. Those who agreed to participate were recruited in the order in which they contacted the research nurses. All subjects underwent physical examination by a medical doctor and laboratory analyses for elevated BP^29^. The medical history and lifestyle habits were documented along with smoking habits, number of cigarettes smoked per day, total smoking duration, and abstinence from smoking in years along with family history for CVD. Alcohol consumption was evaluated as standard drinks (~12 grams of absolute alcohol) per week.

A total of 637 normotensive subjects and never-treated hypertensive patients, aged 19–72 years, were included in the study. They were divided into never smokers (n = 365), present smokers (n = 81) and previous smokers (n = 191). Signed informed consent was obtained from all participants. The study complies with the declaration of Helsinki, and was approved by the ethics committee of the Tampere University Hospital (study code R06086M) and the Finnish Medicines Agency (Eudra-CT registration number 2006-002065-39).

Altogether 247 (39%) of the 637 persons used one or more medications, but the proportions of subjects taking some medication in the never smokers, present smokers and previous smokers did not differ (37.8%, 35.8% and 41.9%, respectively). Seventy-eight female subjects used systemic estrogen, progestin, or their combination (for contraception or hormone replacement therapy), and 1 subject used tibolone. Forty-one subjects were taking antidepressants, 18 antihistamines, 17 inhaled corticosteroids, 15 statins, 13 proton pump inhibitors, while 22 euthyroid subjects were on a stable dose of thyroid hormone. The other medications used by the study population were hypnotics or sedatives (8), low dose acetylsalicylic acid (6), non-steroidal anti-inflammatory drugs (4), antirheumatic agents (4), antiepileptics (3), allopurinol (3), coxibs (3), antipsychotics (2), muscle relaxants (2), varenicline (2), antiviral agents (2), paracetamol (1), carbimazole (1), isotretinoin (1), and alendronate (1). One physically well and symptomless subject was treated with warfarin due to anti-phospholipid syndrome.

### Laboratory analyses

Blood and urine samples were drawn after ~12 hours of fasting. Plasma C-reactive protein, sodium, potassium, glucose, cystatin-C, creatinine, triglyceride, and total, high-density lipoprotein (HDL) and low-density lipoprotein (LDL) cholesterol concentrations were determined using Cobas Integra 700/800 (F. Hoffmann-Laroche Ltd, Basel; Switzerland) or Cobas6000, module c501 (Roche Diagnostics, Basel, Switzerland), insulin using electrochemiluminescence immunoassay (Cobas e411, Roche Diagnostics), and blood cell count by ADVIA 120 or 2120 (Bayer Health Care, Tarrytown, NY, USA). Urine dipstick analysis was made by an automated refractometer test (Siemens Clinitec Atlas or Advantus, Siemens Healthcare GmbH, Erlangen, Germany). Insulin sensitivity was evaluated by calculating the quantitative insulin sensitivity check index (QUICKI)^30^, and glomerular filtration rate (GFR) was estimated using the CKD-EPI creatinine-cystatin C equation^31^.

### Pulse wave analysis

Radial BP and pulse wave were continuously captured from the radial pulsation using a tonometric sensor (Colin BP-508T, Colin Medical Instruments Corp., USA), which was secured on the radial pulse with a wrist band. The radial BP signal was calibrated twice during each 5 minute-period of recording by brachial BP measurements from the contralateral arm. Aortic BP was derived with the SphygmoCor system (SpygmoCor PWMx^R^, AtCor medical, Australia) by means of the validated generalized transfer function^32^. Left ventricular ejection duration, forward wave amplitude (FWA), aortic pulse pressure and reflection time, AIx (augmented pressure/pulse pressure * 100), AIx adjusted to heart rate 75/min (AIx@75), and amplification of pulse pressure and systolic pressure (radial pressure/aortic pressure) were determined.

### Whole-body impedance cardiography

Beat-to-beat heart rate, stroke volume, cardiac output, and pulse wave velocity (PWV) were recorded using whole-body impedance cardiography (CircMon^R^, JR Medical Ltd., Tallinn, Estonia). This method records changes in body electrical impedance during cardiac cycles. Systemic vascular resistance was calculated using the BP signal from the radial tonometry and the cardiac index measured by the CircMon^R^ device. Systemic vascular resistance was calculated by subtracting normal central venous pressure (4 mmHg) from mean arterial pressure and dividing it by cardiac output. Systemic vascular resistance and cardiac output were related to body surface area and presented as indexes – cardiac index, and systemic vascular resistance index (SVRI), respectively. The method and electrode configuration have been previously reported in detail^33,34^.

The stroke volume values measured using CircMon^R^ agree well with 3-dimensional ultrasound^35^. The supine and upright cardiac output values measured with CircMon^R^ agree well with the values measured using thermodilution^33,34^. The PWV values recorded using CircMon^R^ show very good correlations with values measured using ultrasound and the tonometric SphygmoCor method^33,34,36^.

### Experimental protocol

Hemodynamics were recorded in a quiet, temperature-controlled laboratory by trained research nurses^37,38^. Caffeine containing products, smoking or heavy meal were to be avoided for ≥4 hours, and alcohol consumption for >24 hours prior to the studies. The subjects rested supine on the tilt-table with the electrodes placed on body surface, the tonometric sensor on the left radial pulsation, and the oscillometric brachial cuff to the right upper arm. The left arm with the tonometric wrist sensor was abducted to 90 degrees in an arm support, which held the extended arm steady and kept the measurement probes at the heart level both supine and upright.

The actual measurement consisted of one 5-minute period supine and second 5-minute period upright. For the statistical analyses the mean values of each 1-minute period of recording were calculated. The analyses provided information about peripheral and central BP and heart rate^39^, and evaluated large arterial stiffness by measurements of central pulse pressure, FWA, and PWV^21,26,40^. The transit of forward pressure waves in the arterial tree was evaluated by recording the amplification of the systolic pressure and pulse pressure^26,41–43^, and the influence of reflected waves by the variables aortic reflection time and AIx^27,39,44^. Cardiac performance was examined by the evaluations of left ventricular ejection duration, stroke volume, and cardiac output, while resistance arterial tone was estimated by the calculation of systemic vascular resistance^37,38^. Previously, the good repeatability and reproducibility of the measurement protocol has been demonstrated^45^.

### Statistics

The demographic and laboratory data was analysed using analysis of variance (ANOVA), and the homogeneity of variances was tested with the Levene’s test. If variable distribution was skewed, Kruskal-Wallis was applied with Mann-Whitney U-test in the post-hoc analyses (Table 1). The Bonferroni correction was applied in the post-hoc analyses. Haemodynamic differences between the individual groups were examined in supine and upright positions using ANOVA for repeated measures. The analyses were adjusted for age, and for the following variables that presented with significant differences between the groups in univariate analyses: sex, body mass index (BMI), use of alcohol as standard doses per week, LDL cholesterol; and in analyses concerning PWV also for systolic BP. The analyses were not adjusted for triglycerides, since increased plasma triglyceride concentration may represent a true effect of smoking on plasma lipids^9,10^.

**Table 1:** Basic Clinical Characteristics and Laboratory Results.

	Never smoker (n = 365)	Present smoker (n = 81)	Previous smoker (n = 191)
Male/female	164/201	43/38	107/84*
Age (years)	44.2 (0.6)	44.2 (1.3)	46.7 (0.8)
Body mass index (kg/m^2^)	26.2 (0.2)	26.4 (0.5)	28.0 (0.3)*^†^
Office systolic BP (mmHg)	139.6 (1.1)	136.9 (2.4)	144.4 (1.6)^†^
Office diastolic BP (mmHg)	88.7 (0.6)	87.5 (1.4)	91.8 (0.9)*^†^
Cigarettes/day	0	5 [2–12]*	10 [3–19] *^†^
Smoking duration (years)	0	15 [7–25]*	10 [3.0–16.5]*^†^
Total number of cigarettes	0	21900 [7300–87600]*	21900 [4562–79387]*
Smoking abstinence (years)	n.a.	0	10 [3–20]^†^
Alcohol (standard drinks/week)	2.0 [0.0–4.0]	5.5 [2.0–13.0]*	3.0 [1.0–9.5]*^†^
Estimated GFR (ml/min/1.73 m^2^)	99.3 (0.8)	98.5 (1.6)	96.4 (1.0)
Hemoglobin (g/L)	143.0 (0.7)	146.0 (1.2)	145.6 (0.8)
Fasting Plasma			
Sodium (mmol/l)	140.4 (0.1)	140.5 (0.2)	140.3 (0.1)
Potassium (mmol/l)	3.81 (0.01)	3.79 (0.02)	3.81 (0.02)
C-Reactive Protein (mg/l)	1.5 (0.1)	1.8 (0.3)	2.0 (0.3)
Triglycerides (mmol/l)	0.97 [0.68–1.34]	1.18 [0.86–1.75]*	1.18 [0.86–1.58]*
HDL cholesterol (mmol/l)	1.60 (0.02)	1.52 (0.04)	1.54 (0.03)
LDL cholesterol (mmol/l)	2.91 (0.05)	3.21 (0.11)*	3.26 (0.07)*
Glucose (mmol/l)	5.39 (0.03)	5.54 (0.08)	5.54 (0.04)*
Insulin (mU/l)	9.04 (1.15)	8.86 (0.84)	9.02 (0.47)
QUICKI	0.361 (0.002)	0.355 (0.004)	0.352 (0.003)^#^

Results shown as mean (standard error of mean) or median [25^th^ to 75^th^ percentile]; n.a., not applicable; GFR, glomerular filtration rate (CKD-EPI cystatin-C creatinine formula); QUICKI, quantitative insulin sensitivity check index; *P < 0.05 vs. never smoker; ^†^P < 0.05 vs. present smoker (^#^p = 0.059 vs. never smoker).

Linear regression analysis with the enter method was employed to examine the effect of gender and the haemodynamic variables on the level of AIx in supine and upright positions (Table 2), while stepwise linear regression analysis was employed to examine the associations of demographic, laboratory, and haemodynamic variables with AIx (Supplementary Table). For these analyses the skewed distribution of PWV and triglycerides was corrected by lg_10_-transformation, while alcohol consumption was treated as a series of discrete variables that were assigned a score of either 0 or 1; cut-points for women 0, 1–7, 8–14, and above 15 doses per week; for men 0, 1–14, 15–24, and above 25 doses per week, according to the prevailing Finnish Guidelines^46^. Spearman’s correlations (r_S_) were calculated, as appropriate. The results were presented as means and standard errors of the mean (SEM) or median [25^th^ to 75^th^ percentile], and *p* < 0.05 was considered statistically significant. SPSS version 22.0 (IBM SPSS Statistics, Armonk, NY, USA) was used for the statistics.

**Table 2:** Linear regression analysis with the enter method: hemodynamic variables and sex as explanatory variables for augmentation index.

Augmentation index	b	beta	95% confidence interval for b		P value
			Lower	Upper	
Supine, R^2^ = 0.609, p < 0.001					
Constant	7.069		−16.932	31.069	0.563
Male sex	−6.936	−0.291	−8.423	−5.450	<0.001
Systemic vascular resistance index	0.005	0.245	0.003	0.007	<0.001
Lg_10_ of pulse wave velocity	36.760	0.290	28.668	44.851	<0.001
Stroke index	0.230	0.138	0.106	0.353	<0.001
Heart rate	−0.232	−0.187	−0.337	−0.127	<0.001
Ejection duration	0.093	0.154	0.048	0.138	<0.001
Aortic reflection time	−0.361	−0.474	−0.406	−0.317	<0.001
Upright, R^2^ = 0.733, p < 0.001					
Constant	−10.109		−28.205	7.987	0.273
Male sex	−1.867	−0.076	−3.286	−0.448	0.010
Systemic vascular resistance index	0.002	0.086	0.001	0.003	0.005
Lg_10_ of pulse wave velocity	21.012	0.161	14.842	27.183	<0.001
Stroke index	−0.127	−0.047	−0.295	0.041	0.137
Heart rate	−0.089	−0.080	−0.167	−0.010	0.027
Ejection duration	0.328	0.600	0.292	0.365	<0.001
Aortic reflection time	−0.532	−0.454	−0.585	−0.478	<0.001

Variables used: Systemic vascular resistance index, the common logarithm of PWV, heart rate, stroke volume index. Lg_10_, the common logarithm; n = 631 subjects.

## Results

### Study population and laboratory values

The previous smokers had slightly lower proportion of female subjects, while mean age between the study groups did not differ (Table 1). BMI was higher in previous smokers compared to never- and present smokers. In the office systolic BP was ~6 mmHg higher in previous smokers versus present smokers, while diastolic BP was higher in previous smokers compared to never and present smokers. The median number of consumed cigarettes was 21900 in present and previous smokers, while the median abstinence from smoking in previous smokers was 10 years. The weekly intake of alcohol was higher in present and previous smokers than in never smokers, with slightly higher alcohol intake was also observed in the present versus previous smokers, but the average values were well within the limits of moderate drinking in all groups (Table 1). LDL cholesterol level was higher in present and previous smokers, while triglyceride level was higher in present and previous smokers when compared with never smokers. Fasting plasma glucose was slightly higher in the previous smokers versus the never smokers, while QUICKI values were not significantly different between the groups (Table 1).

### Haemodynamic effects associated with present and previous smoking

**Figure 1:**
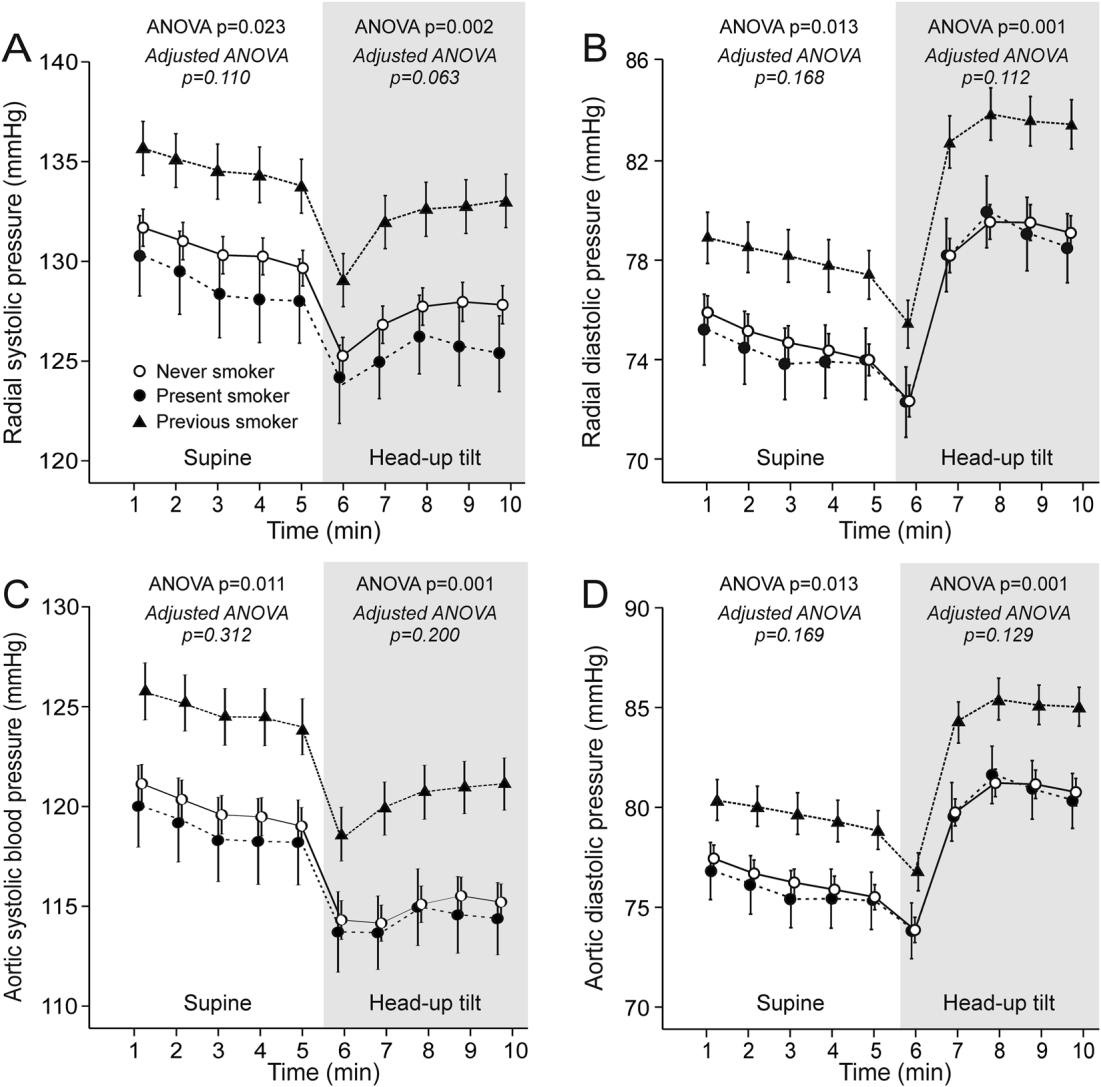
Supine and upright radial systolic (**A**) and diastolic (**B**) blood pressure, and aortic systolic (**C**) and diastolic (**D**) blood pressure in never smokers (n = 365), present smokers (n = 81), and previous smokers (n = 191); mean ± standard error of the mean; ANOVA results from unadjusted analyses (plain text) and from analyses adjusted for age, sex, BMI, LDL cholesterol, and alcohol use (italic) are shown (see Methods).

In unadjusted analyses, radial and aortic systolic and diastolic BP was higher in previous smokers than present smokers and never smokers (Fig. 1A–D). However, when adjusted for age, sex, BMI, LDL cholesterol, and use of alcohol, the differences in BP values between the groups were not significant (Supplementary Fig. A,B). In the text below, only the results of the adjusted analyses are being referred to, while the unadjusted statistics are also shown in the figures.

**Figure 2:**
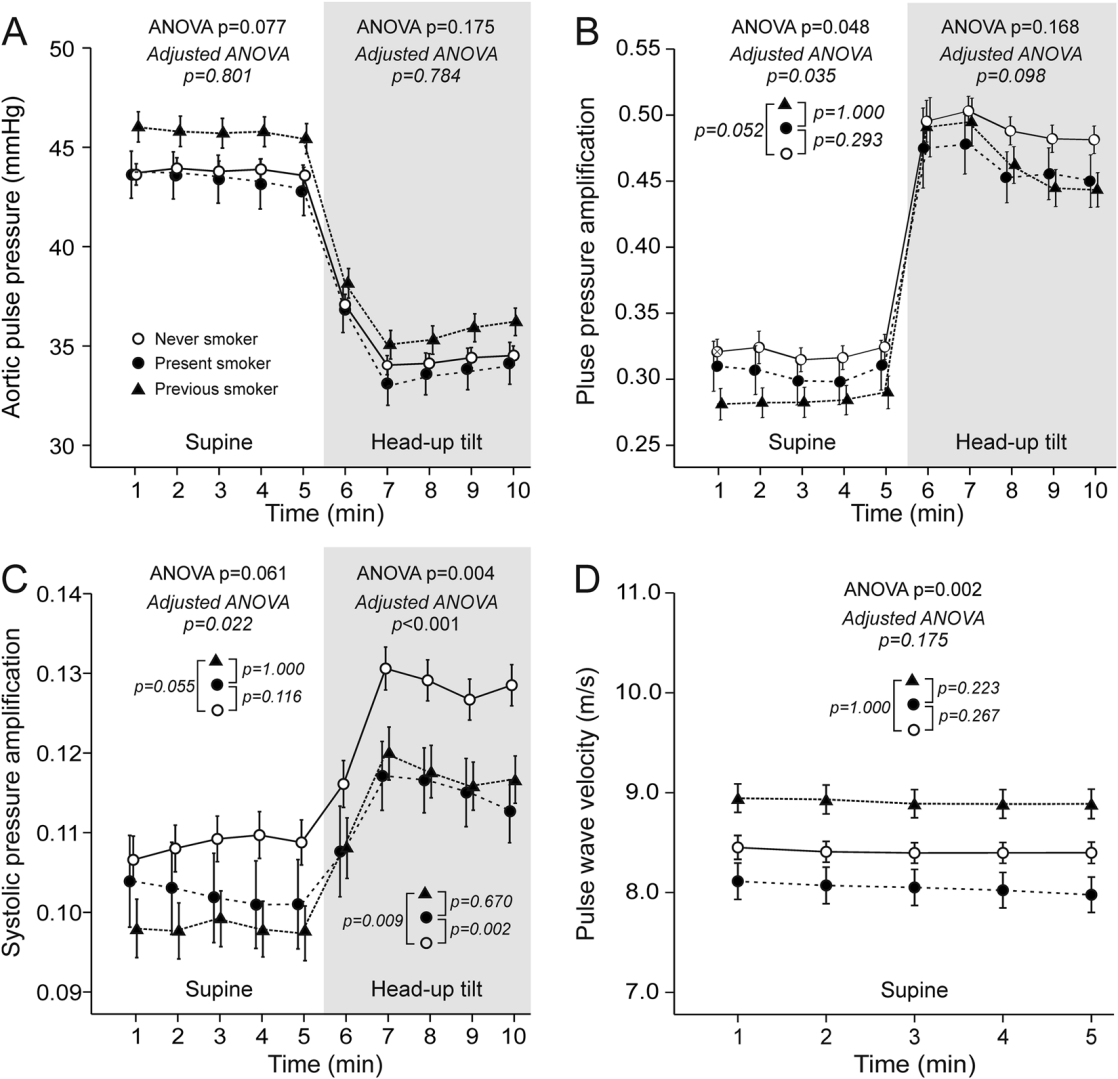
Supine and upright aortic pulse pressure (**A**), pulse pressure amplification (**B**), and systolic amplification (**C**), and supine pulse wave velocity (**D**) in never smokers (n = 365), present smokers (n = 81), and previous smokers (n = 191); mean ± standard error of the mean; ANOVA results from unadjusted (plain text) and adjusted (italic) analyses are shown.

Aortic pulse pressure was not different between the individual groups in either supine or upright position (Fig. 2A). Supine aortic-to-radial amplification of pulse pressure (Fig. 2B) and systolic pressure (Fig. 2C) showed differences in adjusted ANOVA (p = 0.035 and 0.022, respectively), but the differences between individual study groups were not significant. In the upright position, pulse pressure amplification did not differ between the groups (Fig. 2B), while amplification of systolic BP was reduced in the present (p = 0.002) and previous (p = 0.009) smokers versus never smokers (Fig. 2C). No significant differences were found in PWV between the individual study groups in analyses adjusted for age, sex, BMI, LDL cholesterol, use of alcohol, and systolic BP (Fig. 2D).

**Figure 3:**
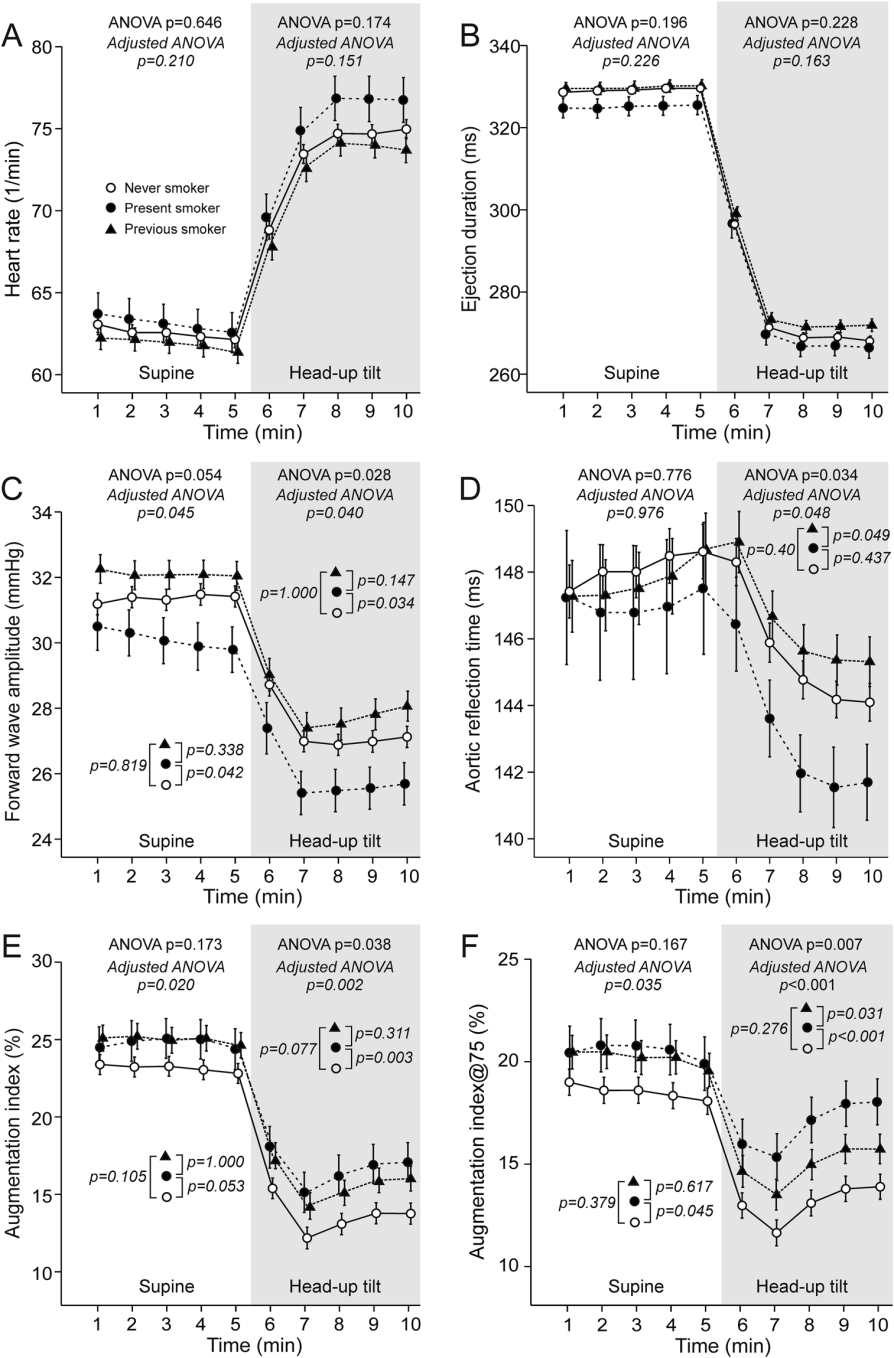
Heart rate (**A**), ejection duration (**B**), forward wave amplitude (**C**), aortic reflection time (**D**), augmentation index (**E**), and augmentation index adjusted to heart rate of 75 beats per minute (**F**) in never smokers (n = 365), present smokers (n = 81), and previous smokers (n = 191); mean ± standard error of the mean; ANOVA results from unadjusted (plain text) and adjusted (italic) analyses are shown.

Neither heart rate (Fig. 3A) nor ejection duration (Fig. 3B) differed between the study groups. Both supine and upright FWA was lower in present smokers than in never smokers (p ≤ 0.042) (Fig. 3C). Supine aortic reflection time was not different between the groups, but was shorter in present smokers than in previous smokers (p = 0.049) during upright position (Fig. 3D).

ANOVA of AIx in the supine position indicated differences (p = 0.020) but the deviations between individual groups were not significant (Fig. 3E). However, heart rate adjusted AIx@75 was increased (p = 0.045) in the supine position in present smokers versus never smokers (Fig. 3F). In the upright position, both AIx and AIx@75 were higher in present smokers than in never smokers (p ≤ 0.003), while AIx@75 was also higher in present smokers than in previous smokers (p = 0.031, Fig. 3E,F, Supplementary Fig. C).

**Figure 4:**
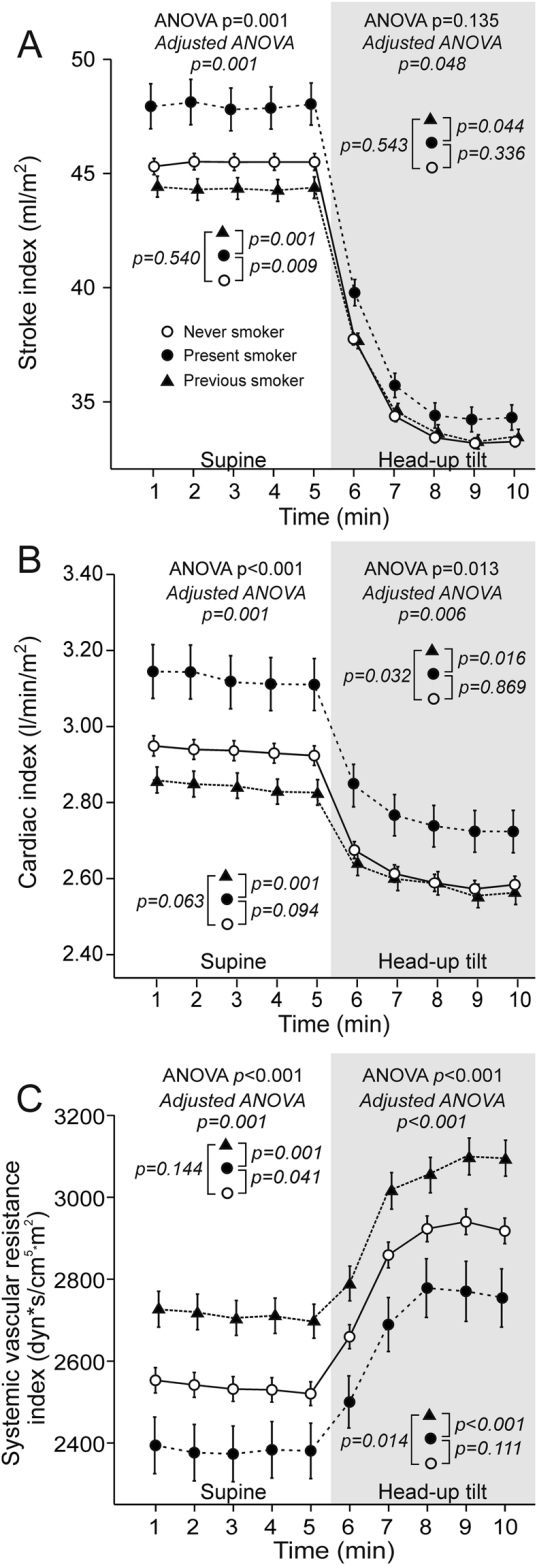
Stroke index (**A**), cardiac index (**B**), and systemic vascular resistance index (**C**) in never smokers (n = 365), present smokers (n = 81), and previous smokers (n = 191); mean ± standard error of the mean; ANOVA results from unadjusted (plain text) and adjusted (italic) analyses are shown.

Supine stroke index was higher in present smokers when compared with never smokers (p = 0.009) and previous smokers (p = 0.001), while upright values were higher in present than in previous smokers (p = 0.044) (Fig. 4A). Cardiac index was increased in present smokers versus previous smokers both supine and upright (p ≤ 0.016), while cardiac index was lower in previous smokers than in never smokers in the upright position (p = 0.032, Fig. 4B, Supplementary Fig. D). When compared with never smokers, supine but not upright SVRI was lower in present smokers (p = 0.041), while upright but not supine SVRI was increased in the previous smokers (p = 0.014). Both supine and upright SVRI was higher in previous smokers versus present smokers (p ≤ 0.001) (Fig. 4C).

#### Results of analyses in subjects not taking medications

Altogether 227 never smokers, 52 present smokers, and 111 previous smokers were without any regular medications. In these subjects, supine AIx@75 was not different between never smokers and present smokers (p = 0.133), while the main findings showing increased AIx (p = 0.007) and AIx@75 (p = 0.005) in the upright position in the present smokers versus never smokers were still detected. The differences in supine stroke index (p = 0.007) and cardiac index (p = 0.038), and upright aortic reflection time (p = 0.038) remained significant between present smokers and previous smokers. However, some deviations were found when compared with the whole study population: Supine SVRI was not higher in previous smokers than present smokers (p = 0.083), but was higher in previous smokers than in never smokers (p = 0.013). Supine stroke index did not differ between present smokers and never smokers (p = 0.213), while upright stroke index did not differ between present smokers and previous smokers (p = 0.063). Upright cardiac index did not show differences between the groups (previous smokers versus never smokers p = 0.074), while supine and upright SVRI were no more different between present smokers and never smokers (p = 1.000 and p = 1.000, respectively). Supine pulse pressure amplification did not differ (p = 0.072), while supine systolic amplification was lower in previous smokers than never smokers (p = 0.022).

### Multivariate analysis about the factors associated with augmentation index

Present smoking increased AIx although it did not reduce heart rate, elevate PWV, or increase systemic vascular resistance, i.e. induce changes in the variables that are most often related to an increase in AIx^36,39,40,47^. Therefore, linear regression analysis was performed to examine the relations of the haemodynamic variables with AIx (Table 2). Due to its powerful confounding, sex was included in the model^36,38,39,48^. These analyses showed that sex and the hemodynamic variables SVRI, PWV, heart rate, ejection duration, and aortic reflection time were significant explanatory variables for AIx (p ≤ 0.027) in both supine and upright positions. Stroke index was a significant explanatory variable for AIx in supine (0 < 0.001) but not in the upright position (p = 0.137). The overall R^2^ values for the model were 0.609 in the supine and 0.733 in the upright position.

Additionally, the relations between demographic variables, smoking status, alcohol intake, laboratory variables, haemodynamic variables, and AIx were examined by the use of regression analysis (Supplementary Table). These analyses also showed that the variables that could explain an increase in AIx in present smokers, i.e. elevated supine stroke index and shorter upright aortic reflection time, were independently associated with AIx. Moreover, present smoking was related with elevated AIx both supine and upright.

## Discussion

Previous reports about the influence of smoking on the level of BP and arterial stiffness have been contradictory^14,15,17,22,49^. Here we examined the haemodynamic effects of smoking using non-invasive recordings of central BP, arterial stiffness, cardiac performance, and systemic vascular resistance. A passive head-up tilt was included, as possible changes in haemodynamics may become more apparent during upright posture^37,38,50^. Many studies have found that smoking increases AIx, but our findings for the first time suggest that AIx may be higher in smokers due to an increase in cardiac stroke volume and shortening of the aortic reflection time.

Smoking has not been associated with consistent effects on BP^14,15,17^. In the present study, higher BP in previous smokers was attributed to the confounding effects of age, sex, BMI, use of alcohol, and LDL cholesterol. In the adjusted analyses, neither present nor previous smoking influenced BP, corresponding to some previous reports^17,51^. This emphasises that the confounding factors must be carefully taken into account in all analyses^52^. Quitting smoking predisposes to increases in weight and BP^19^, while the risk of hypertension increases in previous smokers with increasing years of abstinence^53^.

Smokers may have higher serum cholesterol and triglyceride levels than never smokers^54,55^. Insulin resistance in smokers can alter lipid and lipoprotein metabolism^56^. Previously, smokers had higher triglyceride levels than never smokers in the absence of differences in LDL cholesterol^10^. Smokers also exhibited higher postprandial increases in triglyceride levels than non‐smokers, indicating impaired lipolytic removal capacity^9^. Altogether, smoking promotes atherosclerosis via several mechanisms including changes in blood clotting and lipids, endothelial function, insulin sensitivity, and autonomic tone^7,11–13,54,57^. In our study, the present smokers had higher plasma concentrations of LDL cholesterol and triglycerides than never smokers. In previous smokers LDL cholesterol and triglycerides were also higher than in never smokers, probably due to the increased BMI^11,58^.

Increased PWV is a strong predictor of CVD mortality independent of the level of BP^21^. Early stages of atherosclerosis do not influence the stiffness of the arterial wall, while advanced calcified plaques are associated with increased arterial stiffness^59^. Previously, carotid IMT and plaques were not associated with aortic PWV when adjusted for the confounders age, gender, BP, smoking, and diabetes^60,61^. Thus, aortic stiffness does not predict the severity of carotid atherosclerosis^60,61^. The influence of smoking on PWV remains controversial, and all investigations have not found differences in arterial stiffness between smokers and non-smokers^22,23^. In the present study, PWV did not differ between present smokers and never smokers, while PWV was highest in previous smokers. However, when adjusted for the above confounders, PWV did not differ between the study groups.

The level of AIx, a marker of wave reflections, is influenced by arterial stiffness, heart rate, ventricular ejection duration, body height, BP, systemic vascular resistance, and stroke volume^36,37,39,40,47^. Previous reports have shown that acute, chronic and passive smoking are associated with increased AIx^14,24–28^. However, this has not been attributed to the variables that are known to increase the level of augmentation, like lower heart rate, increased arterial stiffness, or higher systemic vascular resistance^24,26,36,48,49,62^ In our study present smokers had higher upright AIx, and higher supine and upright AIx@75, in the absence of changes in BP, heart rate, ejection duration, PWV, and systemic vascular resistance that could explain an increase in AIx. However, present smokers presented with increased stroke index in supine and upright positions, and decreased upright aortic reflection time versus previous smokers. Both of these factors are associated with higher AIx^36,39,44^. In order to elucidate the haemodynamic determinants of wave reflection, we performed regression analyses of the explanatory variables of AIx. These analyses confirmed that stroke index was an independent determinant of supine AIx, while shorter aortic reflection time was associated with higher AIx both supine and upright. Corresponding to a previous report showing that smoking cessation is associated with reductions in AIx^28^, the level did not differ between previous smokers and never smokers.

Nicotine in tobacco smoke stimulates the sympathetic nervous system^12,63^. In male smokers, nicotine elevated metabolic rate at rest, and increased energy expenditure during light exercise^64^. The present results showed that smoking was associated with increased stroke index in the absence of changes in heart rate. Thus, smoking stimulated the contractile properties of the heart, probably via mechanisms that increase the metabolic rate^64^ and elevate the sympathetic tone^12,63^. Increased stroke index was also translated to higher cardiac output in present smokers versus previous smokers. Previously, current smokers had higher cardiac output than never smokers in an ultrasound-based evaluation^18^.

We found that systemic vascular resistance in the present smokers was lower than in never smokers in the supine position, and lower than in previous smokers in both supine and upright positions. Such haemodynamic changes may be related to the impaired oxygen transport properties of blood during smoking, as carbon monoxide in cigarette smoke increases the levels of carboxyhemoglobin in red cells^13^. Carbon monoxide has also vasodilatory properties^65^. Corresponding to our findings, male smokers presented with vasodilatation in the palmar microvasculature when compared with non-smokers^51^.

Smokers have twice the death rate versus never smokers due to coronary events, while in patients with coronary heart disease the risk of mortality is reduced after 2 years of abstinence from smoking^66^. There is some immediate reduction in CVD risk after smoking cessation^41^, but the period of the remaining increase in risk remains unclear^67,68^. In the present study, upright cardiac output was reduced and systemic vascular resistance was increased in previous smokers versus never smokers. These findings after 10 years of abstinence may represent persistent changes in haemodynamics after the withdrawal of the 10-year-long influence of tobacco smoke on cardiovascular regulation.

In contrast to the increase in PWV and AIx with increasing age, aortic reflection time is only moderately shortened during ageing^44^. In the present study, age correlated strongly with PWV (r_S_ = 0.67) and AIx (r_S_ = 0.57 supine, r_S_ = 0.52 upright), but only moderately with aortic reflection time (r_S_ = −0.31, r_S_ = −0.23 upright) (p < 0.001 for all). In concert with earlier findings^26^, our results showed that upright aortic reflection time was faster in the present smokers than previous smokers. Shorter aortic reflection time provides a possible explanation for the difference in upright AIx@75 between these groups. Although upright AIx and AIx@75 were higher in the present smokers than in never smokers, neither upright aortic reflection time nor upright stroke index differed between these groups. The possibility remains that statistically insignificant changes in the above variables resulted in higher wave reflections in the present smokers. Supporting this view, the relation of stroke index to aortic reflection time was higher in present smokers than in never smokers both supine and upright (0.334 ± 0.007 versus 0.314 ± 0.03 ml/m^2^/ms, p = 0.009; 0.248 ± 0.004 versus 0.237 ± 0.002 ml/m^2^/ms, p = 0.021; respectively).

Aortic-to-brachial pulse pressure amplification reflects arterial compliance in the upper limb, showing reduced values with ageing^42^. Although reduced pulse pressure amplification has been suggested in smokers versus non-smokers^41,43^, the present results did not show differences in this variable between present smokers and never smokers. However, previous smokers presented with impaired amplification of supine and upright systolic pressure. This suggests prevailing differences in the circulation from the aorta to the upper limb in previous smokers, although these findings may also be attributed to the less favourable metabolic profile in this group. Furthermore, upright systolic pressure amplification was impaired in the present smokers versus never smokers. The probable explanation for this is increased augmentation that reduces systolic pressure amplification^26,43^.

This study has some limitations. The results should be interpreted cautiously, as non-invasive measurements were used to evaluate cardiac output, and this requires mathematical processing and simplification of physiology^33^. However, invasive haemodynamic measurements cannot be performed without a clear clinical indication. The present methods have been validated against invasive methods, 3-dimensional ultrasound, and tonometric measurements of PWV^32,33,35,36^. The supine and upright recordings lasted in total for 10 minutes, and this gives a rather narrow window of observation for the study of haemodynamics in humans. The present cross-sectional design does not allow conclusions about causal relationship, and the present findings should be confirmed in follow-up studies. Although all subjects using antihypertensive medications and other medications with direct influences on haemodynamics were excluded, the other medications used by 39% of the study population may have influenced the results. However, the principal findings of the study remained very similar when all subjects taking regular medications were excluded from the analyses.

In conclusion, the present results showed that smoking status had a significant influence on the regulation of cardiac output and systemic vascular resistance in the absence of changes in BP and arterial stiffness. The present smokers presented with hyperdynamic circulation and enhanced wave reflection when compared with previous smokers, while the previous smokers had increased upright systemic vascular resistance and lower cardiac output when compared with never smokers. Finally, our findings suggest that increased AIx in present smokers may be attributed to an increase in stroke volume and shortening of the aortic reflection time.

## Electronic supplementary material

Supplementary figure and table

## Data Availability

Analyses and generated datasets during the current study are not available publicly as our clinical database contains several indirect identifiers and the informed consent obtained does not allow publication of individual patient data. The datasets are available from the corresponding author on reasonable request.
