# Supplementary material for: Effect of present versus previous smoking on non-invasive haemodynamics
Source: Sci Rep. 2018 Sep 11;8:13643. doi: 10.1038/s41598-018-31904-6 (PMC6134124; doi:10.1038/s41598-018-31904-6)
Supplement: Supplementary file 1 — Supplementary figure and table [file 41598_2018_31904_MOESM1_ESM.pdf]

Supplementary Information to *Scientific Reports*

Effect of present versus previous smoking on non-invasive haemodynamics

Manoj Kumar Choudhary[1], Arttu Eräranta[1], Antti J. Tikkakoski[1,2], Heidi Bouquin[1],

Elina J. Hautaniemi[1], Mika Kähönen[1,2], Kalle Sipilä[2], Jukka Mustonen[1,3], Ilkka Pörsti[1,3*]

[1]Faculty of Medicine and Life Sciences, University of Tampere, Tampere; Finland

[2]Department of Clinical Physiology, Tampere University Hospital, Tampere; Finland

[3]Department of Internal Medicine, Tampere University Hospital, Tampere, Finland.

Supplementary Figure. Mean values adjusted for age, sex, BMI, LDL cholesterol, and alcohol use: supine and upright aortic systolic (A) and diastolic (B) blood pressure; cardiac index (C) and augmentation index related to heart rate 75/min (D) in never smokers (n=365), present smokers (n=81), and previous smokers (n=191).

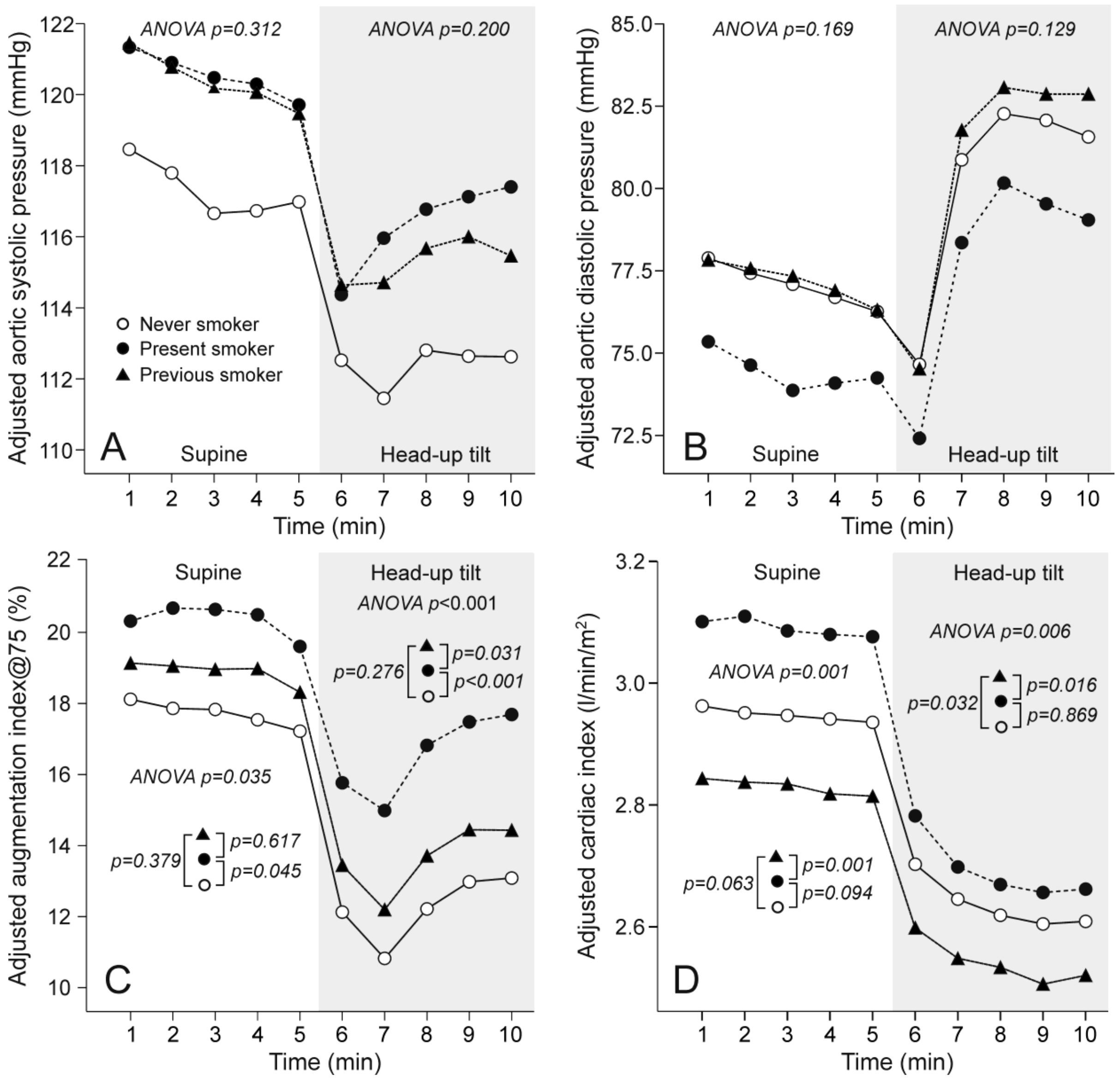

Supplementary Table. Stepwise linear regression analysis of explanatory variables for augmentation index.

Augmentation index	b	beta	95% confidence interval for b		P value
			Lower	Upper	
Supine, $R^2 = 0.697$, $p < 0.001$					
Constant	56.754		32.187	81.321	< 0.001
Age	0.395	0.393	0.344	0.446	< 0.001
Male sex	-3.897	-0.164	-5.701	-2.093	< 0.001
Present smoker	2.292	0.064	0.665	3.920	0.006
Height	-0.210	-0.163	-0.304	-0.116	< 0.001
Systemic vascular resistance index	0.005	0.269	0.004	0.007	< 0.001
Stroke index	0.199	0.120	0.084	0.314	0.001
Heart rate	-0.189	-0.153	-0.283	-0.095	< 0.001
Ejection duration	0.052	0.086	0.011	0.094	0.014
Aortic reflection time	-0.283	-0.371	-0.325	-0.240	< 0.001
Upright, $R^2 = 0.785$, $p < 0.001$					
Constant	6.719		-5.406	18.843	0.277
Age	0.279	0.269	0.237	0.321	< 0.001
Present smoker	2.934	0.080	1.527	4.341	< 0.001
Height	-0.162	-0.122	-0.218	-0.106	< 0.001
Systemic vascular resistance index	0.003	0.139	0.002	0.004	< 0.001
Ejection duration	0.296	0.541	0.272	0.321	< 0.001
Aortic reflection time	-0.455	-0.388	-0.505	-0.405	< 0.001

Variables used: Age, sex, height, weight, categorized smoking, categorized alcohol intake, low density lipoprotein cholesterol, common logarithm of triglycerides, quantitative insulin sensitivity check index, systemic vascular resistance index, common logarithm of PWV, heart rate, stroke volume index; n=631 subjects.
